# Global and Temporal COVID-19 Risk Evaluation

**DOI:** 10.3389/fpubh.2020.00440

**Published:** 2020-08-07

**Authors:** Mudassar Arsalan, Omar Mubin, Fady Alnajjar, Belal Alsinglawi, Nazar Zaki

**Affiliations:** ^1^School of Computer, Data and Mathematical Sciences, Western Sydney University, Sydney, NSW, Australia; ^2^College of IT, UAE University, Al Ain, United Arab Emirates

**Keywords:** COVID-19, inform index, infectious disease vulnerability index, mortality risk evaluation, public health

## Abstract

The COVID-19 pandemic has caused unprecedented crisis across the world, with many countries struggling with the pandemic. In order to understand how each country is impacted by the virus and assess the risk on a global scale we present a regression based analysis using two pre-existing indexes, namely the Inform and Infectious Disease Vulnerability Index, in conjunction with the number of elderly living in the population. Further we introduce a temporal layer in our modeling by incorporating the stringency level employed by each country over a period of 6 time intervals. Our results show that the indexes and level of stringency are not ideally suited for explaining variation in COVID-19 risk, however the ratio of elderly in the population is a stand out indicator in terms of its predictive power for mortality risk. In conclusion, we discuss how such modeling approaches can assist public health policy.

## 1. Introduction

At the end of 2019, a new respiratory tract infection emerged in Wuhan, China. Termed COVID-19, the virus has spread all over the globe, with the World Health Organization (WHO) designating it a pandemic. Highly contagious, the disease has severely impacted economies and elderly populations. Data scientists, epidemiologists and mathematicians are aiming to understand and project the spread of the virus or assess the risk in each country; specifically the risk of deaths is of deep and grave concern. It is widely established that a range of factors prevail, upon which the COVID-19 risk or vulnerability of an individual country depends (1, 2); extending from risk assessment of other viruses and pandemics (3). Therefore, amongst the large volume of work on the impact of COVID-19, a stream of research attempts to decipher the various baseline or constituent factors that could put a nation at risk to COVID-19 (4). These include the study of socio-demographic or economic factors as well as natural elements, such as climate or temperature (5). Typically, such factors are baseline in the sense that they cannot be altered or varied overnight. However, they can pre-empt contingency plans and action points for agencies and organizations enabling countries to be better prepared in response to the virus (6). A region or country at assumed high risk could take timely actions to prepare for and preempt the spread. Prior work has also in relation focused on how different countries compare on their level of risk (7). The wide variety of such indicators ultimately illustrates that selection of appropriate indicators requires clear justification. In prior work, we have also seen the usage of standardized risk indexes developed by large organizations. These indexes are an aggregate of many indicators and factors (8). Such indexes may also play an important role in the evaluation of COVID-19 risk. However, these indexes were in general derived before COVID-19 by considering other viruses and as such their charting of COVID-19 as a pandemic is not immediately clear or established. One may question if these indexes can be readily used to assess COVID-19 risk given that it is acknowledged that COVID-19 is much more contagious than originally thought in comparison to other corona viruses (9). Further, computing the risk of COVID-19 is largely complicated (10)—leading to some efforts to deduce a customized index (11).

In parallel, we also have research focusing on the impact of factors which are more fluid and variable in nature and depend on government intervention and policy (12). These include social distancing measures, travel bans, lockdowns, economic shutdown and much more (13). These can in theory be implemented overnight and in conjunction with the baseline variables provide an effective response strategy. Timely interventions are hence what most government agencies aspire to. Evaluating their effectiveness should also be an integral component of risk modeling (14). Temporal evaluations at key checkpoints are essential to the successful implementation of a response plan keeping in mind non-pharmaceutical interventions, so that scalable strategies can be employed.

While some of the prior work aims to project the efficiency of future actions or predict the spread (15, 16), our research has attempted to evaluate the current or present situation in terms of identifying the mortality risk of COVID-19 on a *global* scale using a wide range of predictors or indicators. Most of the prior work attempts to assess the risk situation on a local scale (17). The aim of any intervention or introduction of a stringency level is to reduce the overall risk. Further, there should be regular and timely determinations to deduce if the interventions are contributing to a lowering of risk. Therefore, in this current paper, we present our analysis on modeling the current and temporal change in COVID-19 risk on a global scale through the incorporation and comparison of not only available and standardized risk indexes but also a temporal factor in terms of the prevailing stringency level. As mentioned prior, these risk indexes do not allow us to reassess the risk when the condition of a country changes (such as when government interventions or lockdowns are activated or softened). Nevertheless, a number of such pre-made indexes are available and it would be worthwhile to compare them in terms of their ability to explain the risk of COVID-19. Therefore, in our research, we compare two standardized indexes in terms of their efficacy to assess the risk and vulnerability of each country toward COVID-19 and also introduce an additional factor in terms of the stringency level of each country.We believe our analysis is a contribution to literature as previous temporal risk assessments for COVID-19 are for specific countries, such as China (18) or South Africa (19).

## 2. Method

Our aim was to discern the risk of COVID-19 on a global yet temporal scale.In order to compute the risk, we wanted to benchmark each country against its prevailing conditions and disease vulnerability as measured by various indicators and predictors.

### 2.1. Index Selection

In Gilbert et al. (8), a range of indexes and their applicability to assess COVID-19 risk are discussed. Two indicators namely the Inform global risk index (20) and the Infectious Disease index (21) were deemed relevant due to their ability to “account” for not only demographic, socio-economic, environmental and political factors but also transmission risk, infrastructure, vulnerability and coping capacity. Hence, we selected the indicators comprised as part of the two indexes as the primary factors of our risk assessment, given that they nicely complimented each other. Their constituent indicators are summarized in a bullet list below. We also recorded the overall value or composite score of the indexes themselves. Data related to these indexes was obtained from their available official documentation (20, 21); including both the constituent indicators and the composite index score. For the Inform global risk index the composite score was available in the official documentation as “Enhanced Inform 2019.” For the Infectious Disease Vulnerability index, the composite score was available in the official documentation as “Overall Score Normed.”

Each of the indicators were normalized on a range of 0–1. We did however notice, that there was no mention of the ratio of elderly population amongst the list of indicators. Proportion of children was included and this may have been due to the focus of the indexes on prior epidemics which were different in their risk demographic. Therefore, we also included an additional static indicator which illustrated the ratio of elderly in the population (above 65 years old), as provided from World Bank. This index was termed as “A65abp” in our data.

- Inform Index
  1. Natural
  2. Human
  3. Hazard and Exposure
  4. Social-Economics Vulnerability
  5. Vulnerable Groups
  6. Vulnerability
  7. Institutional
  8. Infrastructure
  9. Lack of Coping Capacity.
- Infectious Disease Vulnerability Index
  1. Demographic Domain
  2. Health Care Domain
  3. Public Health Domain
  4. Disease Dynamics
  5. Political Domestic
  6. Political International
  7. Economic Domain.

### 2.2. Stringency Level

Each country's response to the emerging threat of COVID-19 has been fluid, dynamic and unique. There is no one size fits all approach. Therefore, representation of the prevailing stringency is important to model in any risk assessment. With the application of stringent measures the risk of future spread should reduce. In order to capture a temporal assessment of variation of risk we utilized the stringency index proposed as the Oxford COVID-19 Government Response Tracker (OxCGRT) (22). This is defined as “a policy stringency index (calculated) by combining 13 policy indicators, including school and workplace closures, travel bans, as well as fiscal policy measures.” To allow for the measures to take effect, and to give increased importance for measures taken earlier rather than later, in our modeling we considered a weighted average stringency level for any day based on a formula provided in literature (4).

### 2.3. Modeling

Our model considered COVID-19 and stringency data between 22-01-20 and 11-05-20 on 2 weeks intervals, namely (02-03-20, 16-03-20, 30-03-20, 13-04-20, 27-04-20, and 11-05-20), such that the model was run for each date in this list for a temporal risk assessment across all countries data (*N* = 156). The data associated with the virus was extracted from the John Hopkins Repository (23). The number of confirmed cases and mortality were normalized per capita based on the population, per one million people and not on the basis of confirmed cases; due to the inaccuracies and irregularities in testing (24).

We ran eight multiple regression models for each of the six dates mentioned. We had two dependent variables: normalized confirmed cases and normalized mortality. Each dependent variable was modeled four times by using the two sets of indicator independent variables in both split and composite form. The four sets of independent variables were the indicators in the Inform Index (9 in total) and those in the Infectious Disease Vulnerability Index (7 in total) as well each of the indexes in their composite form (a single score each). There was no intermixing of indexes as independent variables across each other or in their split or composite form. All regression models further included A65abp (ratio of elderly in the population) and weighted stringency level on that particular date as additional independent variables. The list of 8 regression model types is summarized below.

**For each date in our window of six identified dates**

1. DV = normalized confirmed cases; lm (IV = 9 constituent factors of the Inform Index, A65abp, stringency level)
2. DV = normalized confirmed cases; lm (Enhanced Inform 2019, A65abp, stringency level)
3. DV = normalized mortality; lm (IV = 9 constituent factors of the Inform Index, A65abp, stringency level)
4. DV = normalized mortality; lm (Enhanced Inform 2019, A65abp, stringency level)
5. DV = normalized confirmed cases; lm (IV = 7 constituent factors of the Infectious Disease Vulnerability Index, A65abp, stringency level)
6. DV = normalized confirmed cases; lm (Overall Score Normed, A65abp, stringency level)
7. DV = normalized mortality; lm (IV = 7 constituent factors of the Infectious Disease Vulnerability Index, A65abp, stringency level)
8. DV = normalized mortality; lm (Overall Score Normed, A65abp, stringency level).

Afterwards, the regression predictors were then assessed for relative importance or assigning of weights using the relaimpo package (25). All modeling was carried out in R.

## 3. Results

Initially, we tested for various assumptions of linear regression models. Using residual plots we checked for normality and only minor deviations from normality were observed. None of the predictor variables were considered to be dropped across the four types of regression models; however, we did check for multicollinearity using the measure variance inflation factor (VIF). We realized the importance of this step in particular when we considered the indexes in their split form, as it could be expected that the predictors may possibly be correlated with each other. VIF scores for all 9 predictor variables within the Inform Index were high and beyond tolerance (>5) for both confirmed cases and mortality. A65abp and the stringency level were within an acceptable range (<5). When we used the composite score of the Inform index alongside A65abp and the stringency level, there were no issues whatsoever with respect to multicollinearity. VIF scores for the predictors within the Infectious Disease Vulnerability Index were beyond tolerance for three of them (>5); namely economic domain, political domestic and health care. A65abp and the stringency level were within an acceptable range (<5). When we used the composite score of the Infectious Disease Vulnerability index alongside A65abp and the stringency level, there were no issues whatsoever with respect to multicollinearity. As with the Inform Index, VIF scores mirrored each other across mortality and confirmed cases for the Infectious Disease Vulnerability Index.

Summary of our multiple linear regression models are presented in the form of tables (see Tables 1–**4**). Tables 1, 2 tend to illustrate that the Inform index had slightly higher predictive power for the risk of COVID-19 confirmed cases. Particularly, Table 1 shows that both A65abp and the composite value of the Inform Index have significant predicting power for the risk of COVID-19 confirmed cases. None of the predictor indicators within the Inform index were significant for either of mortality or confirmed cases. Their high multicollinearity also enforces us to focus on the results from the regression model of the composite Inform Index.

**Table 1:** Regression Results for risk of confirmed cases as predicted by Inform Index in both split and composite form; where for *p*-values “***” represents *p* < 0.001 “**” represents *p* < 0.01 and “*” represents *p* < 0.05.

	**Split form**			**Composite form**		
**Date (all dates are 2020)**	***R*^2^**	**Significant *p*-values**	**Top weights**	***R*^2^**	**Significant *p*-values**	**Top weights**
March 2	0.17	Stringency***	Stringency (0.64)	0.16	Stringency***	Stringency (0.84)
March 16	0.30	None	Social economics vulnerability (0.14) Lack of coping capacity (0.13)	0.21	Inform index*, A65abp*	Inform index (0.48), A65abp (0.44)
March 30	0.33	None	Institutional (0.17) Lack of coping capacity (0.15)	0.23	Inform index*, A65abp**	Inform index (0.46), A65abp (0.54)
April 13	0.40	None	Institutional (0.16) Lack of coping capacity (0.16)	0.28	Inform index**, A65abp***	Inform index (0.46), A65abp (0.54)
April 27	0.43	None	Institutional (0.16) Lack of coping capacity (0.17)	0.30	Inform index***, A65abp*	Inform index (0.56), A65abp (0.43)
May 11	0.40	None	Infrastructure (0.13) Lack of coping capacity (0.17)	0.27	Inform index***	Inform index (0.69), A65abp (0.29)

**Table 2:** Regression Results for risk of mortality as predicted by Inform Index in both split and composite form; where for *p*-values “***” represents *p* < 0.001 “**” represents *p* < 0.01 and “*” represents *p* < 0.05.

	**Split form**			**Composite form**		
**Date (all dates are 2020)**	***R*^2^**	**Significant *p*-values**	**Top weights**	***R*^2^**	**Significant *p*-values**	**Top weights**
March 2	0.18	Stringency***	Stringency (0.63), natural (0.15)	0.15	Stringency***	Stringency (0.89)
March 16	0.07	None	A65abp (0.42)	0.06	A65abp**	Inform index (0.12), A65abp (0.87)
March 30	0.16	A65abp*	A65abp (0.39), lack of coping capacity (0.12)	0.14	A65abp***	Inform index (0.17), A65abp (0.82)
April 13	0.28	A65abp**	A65abp (0.30), lack of coping capacity (0.12), institutional (0.13)	0.22	A65abp***	Inform index (0.20), A65abp (0.82)
April 27	0.31	A65abp**	A65abp (0.27), lack of coping capacity (0.14), institutional (0.14)	0.24	A65abp***	Inform index (0.21), A65abp (0.78)
May 11	0.32	A65abp**	A65abp (0.26), lack of coping capacity (0.15), institutional (0.14)	0.24	A65abp***	Inform index (0.21), A65abp (0.77)

Tables 3, 4 highlight that the Infectious Disease Vulnerability Index has lower predictive power than the Inform Index for the risk of both confirmed cases and mortality for COVID-19. Table 4 also highlights the weakness of the Infectious Disease Vulnerability Index to explain COVID-19 mortality risk as none of its constituent predictors or the index itself in its composite form emerged as significant. Extending from our checks of multicollinearity, we re-executed our linear regression model using the split form of the Infectious Disease Vulnerability Index; but dropped the three predictors having high VIF mentioned earlier. In these new models (six each for normalized cases and normalized mortality; six representing the six dates chosen in our temporal analysis), there were no drastic changes in *R*^2^, if anything it further deteriorated. Our R code and all output generated is presented in a documented Supplementary File.

**Table 3:** Regression Results for risk of confirmed cases as predicted by Infectious Disease Vulnerability Index in both split and composite form; where for *p*-values “***” represents *p* < 0.001 “**” represents *p* < 0.01 and “*” represents *p* < 0.05.

	**Split form**			**Composite form**		
**Date (all dates are 2020)**	***R*^2^**	**Significant *p*-values**	**Top weights**	***R*^2^**	**Significant *p*-values**	**Top weights**
March 2	0.09	Stringency*	Stringency (0.38), health care domain (0.16), A65abp (0.18)	0.07	Stringency*	Stringency (0.52), A65abp (0.37)
March 16	0.09	None	Health care domain (0.19), A65abp (0.27)	0.07	A65abp*	Infectious Disease Vulnerability Index (0.19), A65abp (0.66)
March 30	0.24	Public health domain**	Public health domain (0.18), A65abp (0.18), political domestic (0.17)	0.16	A65abp**	Infectious Disease Vulnerability Index (0.30), A65abp (0.66)
April 13	0.29	Public health domain**	Health care domain (0.17), A65abp (0.17), political domestic (0.18)	0.19	A65abp***	Infectious Disease Vulnerability Index (0.36), A65abp (0.63)
April 27	0.29	Public health domain**, health care domain*	Health care domain (0.18), economic domain (0.22), political domestic (0.16)	0.18	A65abp**	Infectious Disease Vulnerability Index (0.43), A65abp (0.56)
May 11	0.30	Public health domain**, health care domain*, economic domain**	Health care domain (0.20), economic domain (0.3)	0.17	Infectious Disease Vulnerability Index*	Infectious Disease Vulnerability Index (0.55), A65abp (0.45)

**Table 4:** Regression Results for risk of mortality as predicted by Infectious Disease Vulnerability Index in both split and composite form; where for *p*-values “***” represents *p* < 0.001 “**” represents *p* < 0.01 and “*” represents *p* < 0.05.

	**Split form**			**Composite form**		
**Date (all dates are 2020)**	***R*^2^**	**Significant *p*-values**	**Top weights**	***R*^2^**	**Significant *p*-values**	**Top weights**
March 2	0.22	Stringency***, political domestic domain**	Stringency (0.56), political domestic domain (0.15)	0.14	Stringency***	Stringency (0.93)
March 16	0.06	None	Health care domain (0.19), A65abp (0.36)	0.05	A65abp*	Infectious Disease Vulnerability Index (0.18), A65abp (0.71)
March 30	0.10	A65abp*	Health care domain (0.16), A65abp (0.39)	0.08	A65abp**	Infectious Disease Vulnerability Index (0.18), A65abp (0.80)
April 13	0.19	A65abp*	Health care domain (0.14), A65abp (0.36), political international (0.14)	0.15	A65abp***	Infectious Disease Vulnerability Index (0.22), A65abp (0.77)
April 27	0.23	A65abp*	Political international (0.15), political domestic (0.14), A65abp (0.34)	0.19	A65abp***	Infectious Disease Vulnerability Index (0.26), A65abp (0.74)
May 11	0.25	A65abp**	Political international (0.16), political domestic (0.15), A65abp (0.33)	0.21	A65abp***	Infectious Disease Vulnerability Index (0.28), A65abp (0.72)

### 3.1. Discussion

In general the response variable variation or *R*^2^ was low for both indexes and for both response variables, where most of the indicators were not integral to the predictive power of the model. The trend of both indexes was similar, as slight improvements in predictive power of the model occurred over time. The inform index did not have any significant constituent indicators within its ranks.

Indicators from within the Infectious Disease Vulnerability Index, such as public health domain were at different times able to explain the variation in risk of COVID-19 confirmed cases across the countries as shown by its significant predictive power. The inform index in its composite form was a stronger predictor for the risk of COVID-19 confirmed cases, whereas the Infectious Disease Vulnerability Index was a significant predictor of the risk of COVID-19 confirmed cases on May-11. This clearly highlights that these indexes if anything, the indexes were slightly more effective in explaining the variation in confirmed cases on a global scale as compared to mortality. The seemingly overall weak results highlight the need of a customized and tailored composite index for risk assessment related to COVID-19, particularly for mortality. Prior literature also identifies the importance of considering COVID-19 as very different from infectious diseases of the past [such as SARS (26)]. Our identified indexes in this paper were conceptualized in the pre-COVID era and hence are finding it difficult to map and predict the risk of COVID-19. Nevertheless, the ratio of elderly (A65abp) as a self-introduced indicator was an important predictor for mortality risk as evidenced by its weight and significance across the models. The mortality risk associated with COVID-19 and the elderly is widely recognized (27) and this association also emerged in our results.

A key incremental novelty of our modeling approach to assess COVID-19 risk was the introduction of a temporal independent variable in the form of the stringency level of each country. Stringency appeared to have predictive power till early March, which is when there was most variation in stringency data. Once the World Health Organization declared COVID-19 as a pandemic around mid March (28), most countries scaled up their stringency levels and it lost any association that it had in modeling the mortality risk of COVID-19. This is exemplified by the standard deviation of weighted stringency level for the entire data set, which went from 11.3 on 02-03-2020 to 12.4 on 16-03-2020. This could also explain the drop or no change in *R*^2^ for our date of 16-03-2020 (for 6 of the 8 scenarios). Prior research has shown stronger outcomes in temporal assessments of COVID-19 toward the start of the outbreak (29). Related research (30) further informs us that controlling the spread and risk of the COVID-19 virus relies more on personal and individual measures, such as social distancing rather than only emphasizing large scale governmental interventions.

## 4. Conclusion

In our analysis, we have attempted to use existing indexes to assess prevailing COVID-19 spread and mortality risk. Our results show that due to the inherent differences primarily related to transmission between COVID-19 and other pandemics of the past, future effort is to be dedicated to design customized indexes once the impact of COVID-19 is understood. Further, the level of stringency that a country had imposed was unable to explain the variation across countries when it came to COVID-19 risk. We discuss how this may have been a by-product of COVID-19 being declared a pandemic around mid March 2020 when most countries increased their stringency levels significantly. Our analysis also confirmed the significant association between the ratio of elderly (or above 65 years old) living in a population and COVID-19 mortality risk as well as between the local prevailing demographics and risk of COVID-19 spread. In addition, future analysis of the like can also focus on regional assessments of risk rather than global determinations which shrink countries to single homogeneous index based indicators. Such analysis can contribute toward a better understanding of public health policy on a regional level, where there are more subtle nuances in the available data. As our results have shown global indexes although meant to discern countries at a world level; the complexities of COVID-19 ultimately create challenges in mapping and projecting its outlook on a global scale.

## Data Availability Statement

Publicly available datasets were analysed in this study. This data can be found here: https://data.worldbank.org/; https://covidtrackerbsg.ox.ac.uk/; https://drmkc.jrc.ec.europa.eu/inform-index; https://www.rand.org/content/dam/rand/pubs/research_reports/RR1600/RR1605/RAND_RR1605.pdf.

## Author Contributions

MA conducted the analysis in R. MA and OM were involved in the initial drafting, conceptualization, identification of indexes, and design of the regression modeling. OM drafted and wrote the paper. In sum, MA and OM have contributed equally to the final product. All authors conceived the study, brainstorming discussions, ideas listing and contributed to the drafting of the final document as well as subsequent revisions during the review process.

## Conflict of Interest

The authors declare that the research was conducted in the absence of any commercial or financial relationships that could be construed as a potential conflict of interest.
